# A long-lever spinal orthosis for idiopathic scoliosis: corrective potential in 10 patients

**DOI:** 10.1186/1748-7161-8-S2-O53

**Published:** 2013-09-18

**Authors:** Brian Dovorany, Mark Morningstar

**Affiliations:** 1 Spine & Posture Center, Green Bay, WI USA

## Background

The long-lever orthosis was designed to treat large translational displacements associated with idiopathic scoliosis. Adding a long-lever system allows the practitioner to affect the spine with a relatively low amount of force, while changing the rotational displacement of scoliosis based upon its effect on the thoracic cage. 

## Purpose

The goal of this study was to determine whether a novel long-lever orthosis has the ability to positively impact idiopathic scoliosis. 

## Methods

A sample of 10 patients, ranging in age from 11 to 16 years, with adolescent idiopathic scoliosis presented to a private chiropractic clinic for evaluation and management. All 10 patients had double major scoliosis curve patterns and were fitted for a long-lever orthosis system. Once in place, scoliosis radiographs were obtained while wearing the orthoses. Outcome measurements included Cobb angle and rotational displacement

## Results

The average baseline Cobb angles were 51° thoracic (range 39-76°) and 31° lumbar (range 23-41°). While wearing the long-lever orthosis system, the thoracic and lumbar Cobb angles decreased to an average of 28° and 27°, respectively. In five of the patients tested, additional improvement in thoracic rotation was observed, by an average of 52% (range 12-97%). No patient tested had an increase in curves or rotation while wearing the long-lever orthosis system. 

## Conclusions and discussion

While wearing a specialized long-lever orthosis system, patients saw their Cobb angles and thoracic rotation decrease. This orthosis may help complement exercise-based scoliosis rehabilitation programs for patients with large translational displacements of the thoracic spine. 
